# Supplementary material for: Predicting the Beneficial Effects of Cognitive Stimulation and Transcranial Direct Current Stimulation in Amnestic Mild Cognitive Impairment with Clinical, Inflammation, and Human Microglia Exposed to Serum as Potential Markers: A Double-Blind Placebo-Controlled Randomized Clinical Trial
Source: Int J Mol Sci. 2025 Feb 19;26(4):1754. doi: 10.3390/ijms26041754 (PMC11855719; doi:10.3390/ijms26041754)
Supplement: Supplementary file 1 [file ijms-26-01754-s001.zip › ijms-3431786-supplementary.pdf]

Predicting the Beneficial Effects of Cognitive Stimulation and Transcranial Direct Current Stimulation in Amnestic Mild Cognitive Impairment with Clinical, Inflammation, and Human Microglia Exposed to Serum as Potential Markers: A Double-Blind Placebo-Controlled Randomized Clinical Trial

Supplementary Information

Figure S1. CONSORT Flow Diagram.

CONSORT 2010 Flow Diagram

CONSORT
TRANSPARENT REPORTING of TRIALS

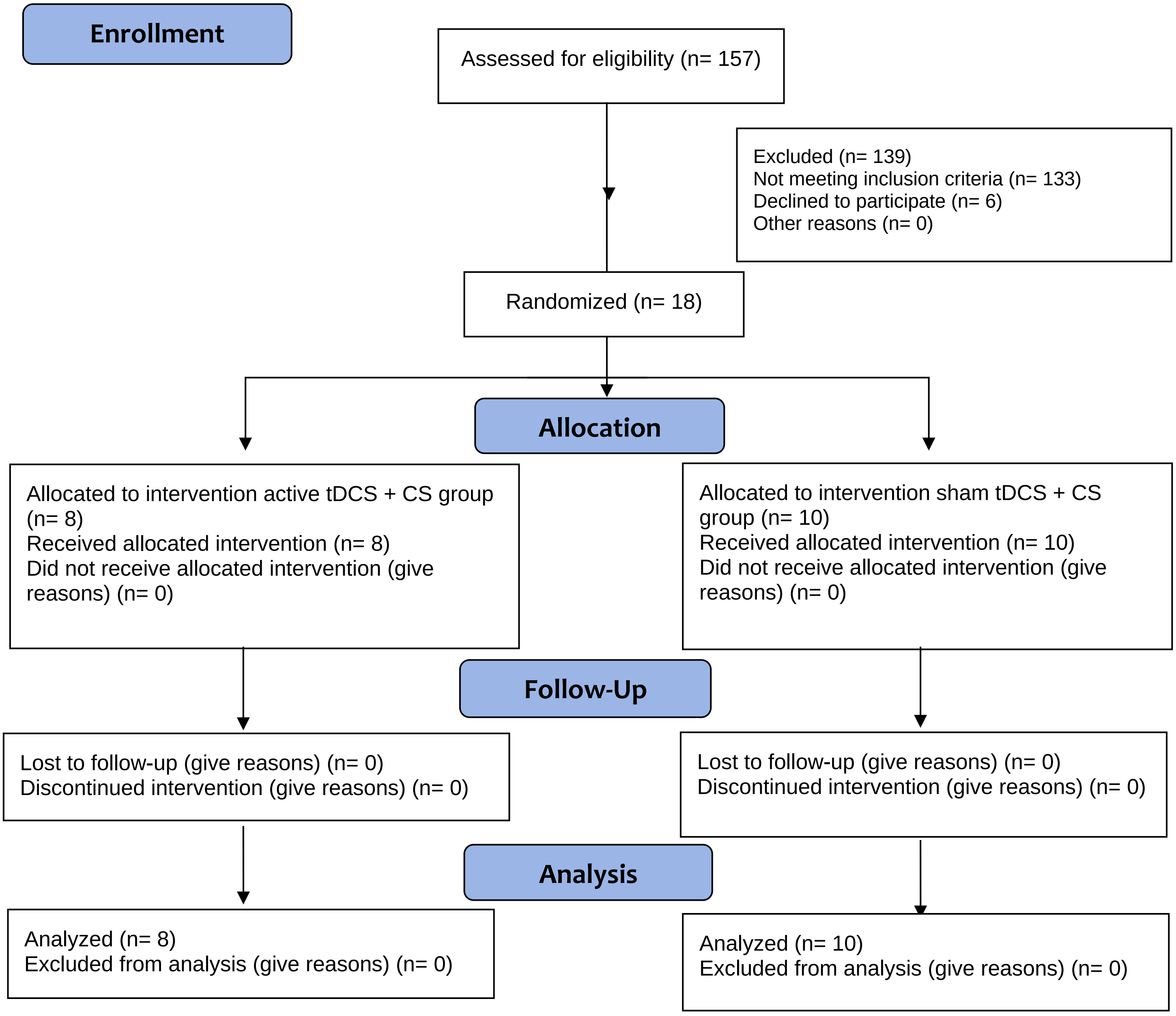

Results

Table S1. Descriptive statistics of standard treatments received by each patient during the study.

	Sham		Active	
	**T0 (n = 10)**	**T1 (n =10)**	**T0 (n = 8)**	**T1 (n =8)**
Medication use, n (%)				
Antidepressants	4 (40%)	-	2 (25%)	-
Anxiolytics	1 (10%)	-	1 (12.5%)	-
Antipsychotic	1 (10%)	-	0 (0%)	-
Antihypertensives	3 (30%)	-	3 (37.5%)	-
Antidiabetics	0 (0%)	-	4 (50%)	-
Insuline	1 (10%)	-	0 (0%)	-
Statins	2 (2%)	-	2 (25%)	-
Hypolipidemics	2 (20%)	-	1 (12.5%)	-
Vasoprotectives	0 (0%)	-	1 (12.5%)	-
Antiplatelets	1 (10%)	-	2 (25%)	-
Spasmolytic	1 (10%)	-	0 (0%)	-
IBP	1 (10%)	-	0 (0%)	-
Hormones	2 (20%)	-	1 (12.5%)	-
Bisphosphonate	1 (10%)	-	0 (0%)	-
Alpha blockers	1 (20%)	-	1 (12.5%)	-

Note: Each medication was monitored by health services independent of the research.

Table S2. Descriptive Statistics of Risk Factors.

	Sham		Active	
	**T0 (n=10)**	**T1 (n=10)**	**T0 (n=8)**	**T1 (n=8)**
Level of education (n, %)				
Incomplete primary education	0 (0%)	-	1 (12.5%)	-
Complete primary education	1 (10%)	-	0 (0%)	-
Incomplete secondary education	1 (10%)	-	0 (0%)	-
High school	1 (10%)	-	0 (0%)	-
Technical education	3 (30%)	-	3 (37.5%)	-
Incomplete bachelor degree	3 (30%)	-	0 (0%)	-
Bachelor degree	1 (10%)	-	4 (50%)	-
Monthly income, mean ± SD	10965 ± 9662.357	-	17438.250 ± 13598.895	-
Income source (n,%)				
Retirement pension	2 (20%)	-	6 (75%)	-
Government support	2 (20%)	-	0 (0%)	-
Family support	1 (10%)	-	0 (0%)	-
Rent	1 (10%)	-	0 (0%)	-
Other	4 (40%)	-	2 (25%)	-
Occupation (n, %)				
Housework	5 (50%)	-	3 (37.5%)	-
Worker	0 (0%)	-	1 (12.5%)	-
Employee	0 (0%)	-	1 (12.5%)	-
Professional	2 (20%)	-	2 (25%)	-
Retired	3 (30%)	-	1 (12.5%)	-
Comorbidities (n, %)				

Hypertension	2 (20%)	-	2 (25%)	-
Diabetes	1 (10%)	-	3 (37.5%)	-
Obesity	1 (10%)	-	1 (12.5%)	-
Smoking	2 (20%)	-	2 (25%)	-
Alcohol consumption	1 (10%)	-	2 (25%)	-
Depression	4 (40%)	-	1 (12.5%)	-
Anxiety	4 (40%)	-	2 (25%)	-
Anthropometry ± SD				
Body weight, kg	68.500 ± 7.783	-	70.850 ± 15.613	-
BMI	28.632 ± 5.470	-	27.541 ± 33.371	-
Physical activity (n, %)				
Intense	5 (50%)	-	3 (37.5%)	-
Moderate	2 (20%)	-	2 (25%)	-
Low	2 (20%)	-	3 (37.5%)	-
Without exercise	1 (10%)	-	0 (0%)	-
Sedentary behavior (+1 hr per day)				
Yes	8 (80%)	-	8 (100%)	-
Social variables				
Live with (n, %)				
Alone	3 (30%)	-	2 (25%)	-
Partner	3 (30%)	-	2 (25%)	-
Partner and sons	1 (10%)	-	3 (37.5%)	-
Other relatives	2 (20%)	-	1 (12.5%)	-
Nursing home	1 (10%)	-	0 (0%)	-
Persons whom patient spend the most time with (n, %)				
Partner	2 (20%)	-	5 (62.5%)	-
Family	6 (60%)	-	2 (25%)	-
Friends	1 (10%)	-	1 (12.5%)	-

Alone	1 (10%)		0 (0%)	
Presence of hobbies (n, %)				
Yes	9 (90%)	-	5 (62.5%)	-
Presence of any outdoor activity (n, %)				
Yes	8 (80%)	-	6 (75%)	-
Self-perceived health (n, %)				
Good	8 (80%)	-	6 (75%)	-
Regular	2 (20%)	-	2 (25%)	-

Note: The risk factors present in patients that could complicate the progression of aMCI, which are considered potentially modifiable are described [13].

Table S3. Outcome Measures: Means and Standard Deviations Across Groups and Time.

Clinical and cognitive parameters

	MoCA Total		MoCA MIS		MoCA Memory	
Time/Group	Active	Sham	Active	Sham	Active	Sham
T0	$\bar{x}$ =22.75 ; σ = 3.06	$\bar{x}$ = 21.80; σ = 2.35	$\bar{x}$ = 9.25; σ = 2.19	$\bar{x}$ = 8.50; σ = 3.44	$\bar{x}$ = 2.25; σ = 1.16	$\bar{x}$ = 2.10; σ = 1.20
T1	$\bar{x}$ = 24.62; σ = 3.07	$\bar{x}$ = 22.80; σ = 2.15	$\bar{x}$ = 10.00; σ = 4.11	$\bar{x}$ = 10.00; σ = 3.94	$\bar{x}$ = 2.75; σ = 1.58	$\bar{x}$ = 2.50; σ = 1.65

	SCIP-S DVL		SCIP-S Fluency		SCIP-S IVL	
	Active	Sham	Active	Sham	Active	Sham
T0	$\bar{x}$ = 5.38; σ = 1.92	$\bar{x}$ = 3.60; σ = 1.65	$\bar{x}$ = 20.75; σ = 5.34	$\bar{x}$ = 18.80; σ = 4.13	$\bar{x}$ = 19.50; σ = 3.96	$\bar{x}$ = 16.10; σ = 2.51
T1	$\bar{x}$ = 5.62; σ = 2.45	$\bar{x}$ = 4.10; σ = 1.66	$\bar{x}$ = 22.25; σ = 2.31	$\bar{x}$ = 18.10; σ = 3.54	$\bar{x}$ = 19.38; σ = 5.24	$\bar{x}$ = 16.10; σ = 3.28

	SCIPS SP		SCIP-S WM		SCIP-S Total	
	Active	Sham	Active	Sham	Active	Sham
T0	$\bar{x}$ = 6.75; σ = 2.25	$\bar{x}$ = 7.30; σ = 1.95	$\bar{x}$ =16.12; σ = 3.56	$\bar{x}$ = 14.30; σ = 2.83	$\bar{x}$ = 57.25; σ = 14.88	$\bar{x}$ = 50.20; σ = 11.12
T1	$\bar{x}$ = 8.00; σ = 2.33	$\bar{x}$ = 7.10; σ = 2.13	$\bar{x}$ = 15.88; σ = 3.52	$\bar{x}$ = 14.60; σ = 5.02	$\bar{x}$ = 59.38; σ = 13.08	$\bar{x}$ = 47.50; σ = 12.78

Biological and microglial parameters

	IL.6		BDNF		FK	
Time/Group	Active	Sham	Active	Sham	Active	Sham
T0	$\bar{x}$ = 94.96; σ = 15.34	$\bar{x}$ = 96.86; σ = 18.31	$\bar{x}$ = 195.05 ; σ = 21.53	$\bar{x}$ = 208.73; σ = 21.72	$\bar{x}$ = 3.31; σ = 0.33	$\bar{x}$ = 3.40; σ = 0.28
T1	$\bar{x}$ = 79.25; σ = 19.96	$\bar{x}$ = 73.61; σ = 16.94	$\bar{x}$ = 269.96; σ = 12.55	$\bar{x}$ = 270.23; σ = 8.89	$\bar{x}$ = 3.20; σ = 0.57	$\bar{x}$ = 3.27; σ = 0.32

	PRO 3		PRO 20		VC 3		VC 20	
	Active	Sham	Active	Sham	Active	Sham	Active	Sham
T0	$\bar{x}$ = 72.57; σ = 0.86	$\bar{x}$ = 72.60; σ = 1.32	$\bar{x}$ = 74.55; σ = 1.20	$\bar{x}$ = 75.13; σ = 1.55	$\bar{x}$ = 55.13; σ = 0.92	$\bar{x}$ = 54.95; σ = 1.30	$\bar{x}$ = 73.07; σ = 1.29	$\bar{x}$ = 72.83; σ = 2.07
T1	$\bar{x}$ = 82.56; σ = 0.92	$\bar{x}$ = 80.74; σ = 1.41	$\bar{x}$ = 81.24 ; σ = 0.84	$\bar{x}$ = 81.00; σ = 1.28	$\bar{x}$ = 63.78; σ = 1.14	$\bar{x}$ = 63.16; σ = 0.64	$\bar{x}$ = 80.97; σ = 1.47	$\bar{x}$ = 80.37; σ = 1.27

Table S4. Factor Loadings Matrix.

Eigenvalues and Eigenvectors

Package used : agricolae; PB-Perfect

	PC1	PC2	PC3	PC4	PC5	PC6	PC7	PC8	PC9	PC10	PC11	PC12	PC13	PC14	PC15
Eigen values and Variance %															
Eigen Value	5.85	2.80	1.62	1.08	0.85	0.60	0.57	0.51	0.45	0.25	0.16	0.12	0.06	0.04	0.04
Variance %	39.00	18.65	10.83	7.23	5.68	3.99	3.83	3.38	2.99	1.65	1.09	0.77	0.39	0.29	0.23
Cumulative Variance %	39.00	57.65	68.48	75.71	81.39	85.37	89.21	92.59	95.58	97.23	98.32	99.09	99.48	99.77	100.00
Eigenvectors															
MoCA.MIS	-0.20	-0.24	-0.41	-0.12	0.30	-0.22	0.43	-0.06	-0.43	0.19	-0.37	-0.11	0.18	0.05	-0.03
MoCA.Total	-0.21	-0.27	-0.22	0.22	0.39	-0.11	-0.08	0.50	0.49	0.28	0.19	0.06	-0.05	0.02	0.08
MFEQ	0.17	0.06	0.45	-0.48	0.30	0.29	0.22	0.28	0.19	0.07	-0.35	0.21	0.14	0.08	-0.02
CGI.S	0.27	-0.09	0.22	-0.25	-0.26	-0.66	-0.18	0.08	-0.06	0.49	-0.02	0.03	-0.11	0.10	0.03
SCIP.S.IVL	-0.08	-0.50	0.03	0.02	0.06	-0.08	-0.52	-0.22	0.05	-0.30	-0.42	0.35	0.09	-0.05	0.11
SCIP.S.VF	-0.07	-0.37	0.21	0.38	-0.43	0.44	0.17	0.02	-0.08	0.46	-0.15	0.05	0.03	0.09	0.04
SCIP.S.DVL	-0.11	-0.42	0.17	-0.39	0.28	0.24	-0.14	-0.33	-0.14	0.16	0.48	-0.21	-0.19	0.01	-0.12
SCIP.S.Total	-0.02	-0.40	0.43	0.16	-0.06	-0.28	0.32	0.29	-0.07	-0.50	0.11	-0.30	0.03	0.01	-0.06
IL.6	0.28	0.11	0.11	0.27	0.34	0.15	-0.41	0.36	-0.55	0.07	-0.11	-0.22	-0.11	-0.09	0.07
BDNF	-0.36	0.16	0.13	-0.03	-0.04	0.00	-0.22	-0.09	0.29	0.07	-0.44	-0.66	-0.13	0.16	-0.11
CX3CL1	0.15	0.14	0.35	0.47	0.46	-0.20	0.16	-0.52	0.14	0.16	0.01	0.05	0.12	0.08	0.02
PRO3	-0.38	0.13	0.18	0.08	0.01	-0.10	-0.06	0.08	-0.13	0.10	-0.02	0.28	-0.06	-0.36	-0.74
PRO20	-0.38	0.17	0.11	0.03	0.03	-0.03	-0.11	0.09	-0.25	-0.09	0.14	0.26	0.01	0.80	0.02
VC3	-0.37	0.13	0.21	-0.09	-0.05	-0.05	-0.16	0.05	-0.10	0.12	0.19	-0.10	0.70	-0.28	0.35
VC20	-0.38	0.12	0.19	-0.03	0.05	-0.07	0.19	-0.02	-0.11	0.00	-0.08	0.18	-0.60	-0.29	0.52

Source: https://medium.com/@victorallan

Note: This table shows the relationship between the principal components and original variables.

Supplementary Methods and Materials

Study Recruitment

Participants aged 60 to 75, identified as being at risk of developing aMCI, were recruited through in-clinic outreach at the Instituto Nacional de Psiquiatría Ramón de la Fuente Muñiz in Mexico City, as well as the Geriatric Clinic of Coyoacán. Additional recruitment efforts were supported by social media advertisements. This study presents the ongoing progress of a double-blind, sham-controlled randomized clinical trial (RCT). The study follows a pre-test, post-test design with control groups and is divided in two phases. In the first phase, the active transcranial direct current stimulation (tDCS) combined with cognitive stimulation (CS) is compared to sham tDCS combined with CS.

Sample size calculation

The sample size was calculated by using the G*Power 3.1 program (citation). A medium-sized effect size of 0.375 was considered to the estimation. A repeated measures ANOVA (between-subjects effect) was applied, with 80% power and alpha of 0.05, two groups, and three measurements. Based on this parameters, the required sample size for this phase is 40 patients, with 20 participants per group.

Supplementary Recruitment Criteria. Clinical Evaluation

a) Diagnostic Process Overview

The diagnostic assessment of older adults, whether cognitively healthy, with mild cognitive impairment (MCI), or with dementia, was based on the exploration and clinical history of the patient conducted by an experienced clinician. This process is initiated with directed questions aimed at identifying any cognitive concerns expressed by the patient or by an informant present during the evaluation, concomitant with the observation of the performance of the patient by the clinician to infer a preliminary diagnosis based on the collected information [1-3].

To confirm cognitive alterations, it was considered whether they fell outside the normal range for age and educational level, assessed using the MoCA. The cutoff points considered for dementia were from 0 to 18 points, for MCI from 19 to 24 points, and for normality from 25 to 30 points. If the score was less than 30 and the subject had 12 or less years of education, one point was added to the total score [4,5].

The evaluating clinician also inquired about performance in complex tasks and used the Lawton & Brody Instrumental Activities of Daily Living Scale (Lawton & Brody IADL) and Katz Index of Independence in Activities of Daily Living (Katz ADL Index) to determine the preservation of independence, aiding in distinguishing between MCI and dementia.

To classify the diagnostic impression among cognitively healthy individuals, those with MCI, or dementia, findings were documented, and clinical judgment was employed. It is important to highlight the significance of this, as it is crucial to recognize that the application of these criteria is challenging due to the need to understand the level of individual functioning in their current stage of life. Having said that, we must acknowledge that detection allows us to identify a clinical syndrome that evidences cognitive decline, enabling us to subsequently carry out a diagnostic approach [1,5,6].

b) Candidate Selection and Additional Test:

Candidates with cognitive alterations as evidenced by MoCA scores below 25, with memory deficits, and who could or could not present difficulties in other cognitive domains -whether reported by the patient or an informant- and who also demonstrated preserved functional independence were invited to continue participating. Furthermore, the cognitive deficits should not be better explained by another condition, such as depression. Those who agreed to continue with the evaluation underwent laboratory tests free of charge (funded by INPRFM): complete blood count, blood chemistry, lipid profile, serum electrolytes, thyroid profile, folic acid, vitamin B12, and general urine test. Once the results were available, if any abnormalities that could condition cognitive failures were found, they were advised to see a specialist and return if the failures persisted.

c) Comprehensive Evaluation

For those with normal laboratory results, anamnesis was conducted by the Psychogeriatric (RAL). This included assessing onset and evolution of cognitive failures, any visual and auditory difficulties through interviews, family history of dementia, treatments, support network, socio-family assessment, global clinical impression. These processes aimed to rule out factors that could influence the manifestations of aMCI, or to verify the presence of cognitive impairments.

The following instruments were used to collect the information: Screening for Cognitive Impairment in Psychiatry (SCIP-S) form 1, Hachinski Ischemic Score (HIS), Charlson Comorbidity Index (CCI), Memory Failures of Everyday Questionnaire (MFEQ), Cognitive Reserve Questionnaire (CRIq), Geriatric Depression Scale (GDS), Neuropsychiatric Inventory (NPI), Clinical Global Impression-Severity (CGI-S), Clinical Global Impression–Improvement (CGI-I).

d) Neuropsychological Assessment

If the impression of aMCI was consistent, a comprehensive evaluation was conducted, as the diagnosis of aMCI requires a neuropsychological assessment with standardized tests showing that the individual cognitive performance is 1 to 1.5 standard deviations below the expected range for their age group [2]. This evaluation included the Integrated Neuropsychological Exploration Program-Barcelona Test (PIEN), NEUROPSI Attention and Memory, F-A-S Test, Stroop Color-Word Test, Trail Making Test (TMT) versions A and B, and Vocabulary subtest of the Wechsler Adult Intelligence Scale IV (WAIS-IV).

Table S5. Study Criteria.

**Inclusion**
• Age between 60-75 years.<br>• Any sex.<br>• Speak Spanish fluently.<br>• ≥ 3 years of education.<br>• Diagnosis of aMCI established according to the modified Petersen diagnostic criteria, MoCA (19-24 points) with evidence of MCI with respect to a level prior to his performance in memory. In addition to the concern of the patient, clinician or a family member of memory failures, it was documented by standardized neuropsychological tests.<br>• If receiving treatment with pharmacotherapy, to complete at least 3 months of therapy before inclusion.<br>• Clinical stability of other comorbidities. With evidence from clinical history and laboratory that the cognitive impairments are not a direct physiological consequence of another medical condition such as diabetes mellitus type, arterial hypertension, dyslipidemia, infections, thyroid disease, vitamin deficiencies, laboratories: complete blood count, blood chemistry, lipid profile, serum electrolytes, thyroid profile, folic acid, vitamin B12 and general urinalysis. Once the results are in, if altered data are found that lead to cognitive failures, they will be instructed to go to the doctor to resolve it and return if the failures persist.
**Exclusion (all)**
• History of stroke and/or focal neurological symptoms.<br>• History of epilepsy.<br>• History of neurological or brain surgery, stroke, brain tumour, aneurysm, severe head injury or other significant neurological diseases.<br>• Severe psychiatric disorders such as: psychosis, bipolar, major depression, drug abuse, etc.<br>• Patients that do not fulfill the security measures for tDCS.<br>• Uncorrected sensory perception disturbances.
**Elimination (all)**
• Desire to terminate their participation by the patients or caretaker.<br>• Changes in the dosage in the pharmacotherapy.<br>• Severe adverse effects of the tDCS.<br>• Worsening of behavioral and/or cognitive symptoms.

Note: amnestic mild cognitive impairment (aMCI); Montreal Cognitive Assessment (MoCA); transcranial direct current stimulation (tDCS).

Table S6. Cognitive Stimulation Program Structure.

Activity	Duration	Materials
Relaxation	10 minutes	Audio and guided readings with deep breathing and muscle tension reduction techniques
Activation	5 minutes	Oral activity workbook from the program
Task review	10 minutes	Worksheets
Start of cognitive stimulation activities with paper and pencil	55 minutes	Manual, pencils, erasers
Closing and task assignment to be presented in the next session	10 minutes	Detachable worksheets from the task section

Table S7. Content per Cognitive Stimulation Session.

Session	Contents
Session 1	Relaxation, instructions, attention, daily life, visualization, calculation
Session 2	Perception, categorization, visualization, association, verbalization, daily activities
Session 3	Visualization, verbalization, association, daily activities
Session 4	Attention, instructions, association
Session 5	Attention, language
Session 6	Memory, attention, daily life
Session 7	Daily life, temporal orientation, daily life, planning, calculation
Session 8	Attention, instructions, planning, hobbies, name recall
Session 9	Everyday forgetfulness, perception

Note: Each content area is addressed through different activities. For instance, "attention" includes working with graphic, numerical, and alphabetical stimuli; in some activities, combined stimuli are presented. For this reason, certain topics appear more than once throughout the 9 sessions, with the aim of diversifying and increasing complexity.

Clinical Assessments Details

The following instruments were used in the overall clinical trial:

- **Sociodemographic Questionnaire:** This brief questionnaire collects patient information such as age, education level, socioeconomic status, marital status, treatment history, and employment status. It also includes clinical characteristics, such as family history of dementia or MCI, anthropometric measurements, current medications, and history of alcohol, tobacco, or substance use. Additionally, it assesses the presence of other diseases and incorporates laboratory screenings, including a complete blood count, serum electrolytes, blood chemistry, lipid profile, kidney and liver function tests, thyroid profile, folic acid, vitamin B12, and general urinalysis.

- **Montreal Cognitive Assessment (MoCA):** The MoCA is a screening tool that evaluates global cognitive function, including short-term memory, visuospatial skills, executive function, attention, concentration, language, and orientation. This instrument is considered the "gold standard" for assessing global cognitive functioning in aMCI. In the memory section, the MoCA calculates the Memory Index Score (MoCA-MIS), which includes both free and cued recall. This score has been identified as a useful indicator for distinguishing amnestic mild cognitive impairment (aMCI) from normal cognition in older adults [7].

- **Screening for Cognitive Impairment in Psychiatry (SCIP-S):** The SCIP-S provides a quick assessment of cognitive deficits associated with psychiatric conditions. Although it is not intended for diagnostic purposes, it measures levels of cognitive performance. The tool includes three parallel forms, allowing for repeated assessments, and evaluates working memory, immediate and delayed verbal learning, verbal fluency, and processing speed [8].

- **Memory Screening:** This questionnaire assesses the frequency and severity of memory lapses in daily life. It evaluates categories of forgetfulness, including "Speaking, Reading, and Writing," "Names and Faces," "Actions," and "Learning New Things." The responses reflect how adults experience their everyday memory function [9].

- **Clinical Global Impression Scale (CGI):** The CGI is a standardized descriptive scale that provides qualitative insights into the patient's clinical condition, evaluating both the severity of the condition (CGI-S) and improvements following therapeutic interventions (CGI-I). The scale is completed by the evaluator, not the patient [10].

Study Timeline

Patients were assessed by RAL and a neuropsychologist (Figure S1). At Visit 1, pre-selected patients arrived for a clinical assessment interview by RAL to confirm that they met criteria. At Visit 2, labs were performed. Neuropsychological assessments were taken at Visit 3. Following interpretation of the neuropsychological assessment results and clinical findings, we initiated the double-blind sham-tDCS plus CS intervention phase (see below). Patients attended regularly scheduled sessions (active or sham tDCS) for 15 days over a 3-week period and received a total of 15 sessions of active or sham tDCS and 9 CS. After 3 weeks (T1), they underwent a clinical assessment and repeated blood sampling and excitability paradigm, marking the end of this phase and the beginning of the maintenance phase, which is not yet reported in this manuscript. Blinding (active vs. sham) was applied to the participants and was open only to the data analyst, not to the rest of the research team.

Statistical Analysis Detailed

All data analyses were carried out in the free software programming language RStudio version 2023.06.2+561 [11] (R Core Team, 2023). Demographic and clinical data of participants were analyzed using descriptive statistics. Subsequently, given the characteristics of the sample, the Shapiro-Wilk normality test was performed to determine the homogeneity contrast between distributions. Since the assumptions of normality and homoscedasticity were not met, it was decided to use non-parametric statistics. For comparisons of age and years of schooling (scalar variables) the Mann-Whitney U test was used, while for sex, occupation, marital status and who lives with (nominal variables) the Fisher exact test was used.

For cognitive variables, the Shapiro-Wilk test showed that the data did not follow a normal distribution with a statistical significance of $p < 0.05$, so it was decided to use a useful approach when numerical or ordinal variables are not normally distributed. The tool is called ARTool (Align-and-rank data for nonparametric factorial ANOVA) in RStudio, which implements an aligned rank transform to run nonparametric analysis of variance in factorial models with fixed and random effects (or repeated measures), applying a factorial ANOVA for each response variable (Kay et al., 2021; Wobbrock et al., 2011).

The response variable was the scores on the MoCA cognitive tests (final score, MoCA-MIS, and memory domain score). For the SCIP-S test, raw scores were considered for the domains of immediate verbal learning (AV-I), delayed verbal learning (AV-D), working memory (WM), verbal fluency (VF), and processing speed (PS) and total score (SCIP-Total).

The fixed factors were group and time, each with two levels (active, sham) and (T0, T1). A grouping term (ID or subject number) was included since there are two measurements of the response variable per patient. This term was used by the package to determine the type of model to be run. When a grouping term was presented, a mixed effects linear model was run on the data.

ARTool specifies that grouping terms do not participate in the transformation but are included in the model when performing the factorial ANOVA. In this way, the effect of each factor (group and time) was evaluated, as well as their interaction according to the two measurements for each subject. It was important to be careful when interpreting the results, since only the main effect or interaction for which the response variable was aligned and classified was taken into account. That is, for each response variable, the result of each of the three ANOVAs performed was considered, to obtain three results in total.

The effect size was calculated with partial eta squared (η^2), a measure of association that describes the proportion of the total variation explained by a predictor variable, after partially excluding the variance of other predictor variables from the total variance without error. For their interpretation, the following values are used: 0.01 – 0.059 represent small effect sizes, 0.06 – 0.0139 medium effect sizes and 0.14 – onwards large effect sizes [12]. In this work, the value of η^2 was calculated from the F value and the degrees of freedom of the resulting interaction for each response variable.

Supplementary References

1. Petersen, R.C. Mild Cognitive Impairment. Continuum 2016, 22, 404–418.
2. Petersen, R.C.; Lopez, O.; Armstrong, M.J.; Getchius, T.S.D.; Ganguli, M.; Gloss, D.; Gronseth, G.S.; Marson, D.; Pringsheim, T.; Day, G.S.; et al. Practice guideline update summary: Mild cognitive impairment: Report of the Guideline Development, Dissemination, and Implementation Subcommittee of the American Academy of Neurology. *Neurology* **2018**, *90*, 126-135, doi:10.1212/WNL.0000000000004826.
3. Jack, C.R., Jr.; Bennett, D.A.; Blennow, K.; Carrillo, M.C.; Dunn, B.; Haeberlein, S.B.; Holtzman, D.M.; Jagust, W.; Jessen, F.; Karlawish, J.; et al. NIA-AA Research Framework: Toward a biological definition of Alzheimer's disease. *Alzheimers Dement* **2018**, *14*, 535-562, doi:10.1016/j.jalz.2018.02.018.
4. Nasreddine, Z.S.; Phillips, N.A.; Bedirian, V.; Charbonneau, S.; Whitehead, V.; Collin, I.; Cummings, J.L.; Chertkow, H. The Montreal Cognitive Assessment, MoCA: a brief screening tool for mild cognitive impairment. *J Am Geriatr Soc* **2005**, *53*, 695-699, doi:10.1111/j.1532-5415.2005.53221.x.
5. Palacios García, A.A. Validez y confiabilidad del Montreal Cognitive Assessment (MoCA) en su versión traducida al español para el cribaje del deterioro cognitivo leve en adultos mayores. Universidad Nacional Autónoma de México, México (UNAM), 2015.
6. Canevelli, M.; Grande, G.; Lacorte, E.; Quarchioni, E.; Cesari, M.; Mariani, C.; Bruno, G.; Vanacore, N. Spontaneous Reversion of Mild Cognitive Impairment to Normal Cognition: A Systematic Review of Literature and Meta-Analysis. *J Am Med Dir Assoc* **2016**, *17*, 943-948, doi:10.1016/j.jamda.2016.06.020.
7. Kaur, A.; Edland, S.D.; Peavy, G.M. The MoCA-Memory Index Score: An Efficient Alternative to Paragraph Recall for the Detection of Amnestic Mild Cognitive Impairment. *Alzheimer Dis Assoc Disord* **2018**, *32*, 120-124, doi:10.1097/WAD.0000000000000240.
8. Pino, O.; Guilera, G.; Rojo, J.; Gomez-Benito, J.; S.E., P. *SCIP-S,Screening del Deterioro Cognitivo en Psiquiatría*; Madrid, 2014.
9. Montejo, P.; Montenegro, M.; Sueiro-Abad, M.; Huertas, E. Cuestionario de Fallos de Memoria de la Vida Cotidiana (MFE). Análisis de factores con población española. **2014**, doi:10.6018/analesps.30.1.131401.
10. Kadouri, A.; Corruble, E.; Falissard, B. The improved Clinical Global Impression Scale (iCGI): development and validation in depression. *BMC Psychiatry* **2007**, *7*, 7, doi:10.1186/1471-244X-7-7.
11. Team, R.C. A Language and Environment for Statistical Computing Software. *R Core Team* **2023**.
12. Adams, M.A.; Conway, T.L.I.E.o.Q.o.L.a.W.-B.R. In Encyclopedia of Quality of Life and Well-Being Research (pp. 1965–1966). Springer Netherlands. *Springer Netherlands* **2014**, 1965–1966, doi:doi.org/10.1007/978-94-007-0753-5_918.
13. Livingston, G.; Huntley, J.; Liu, K.Y.; Costafreda, S.G.; Selbaek, G.; Alladi, S.; Ames, D.; Banerjee, S.; Burns, A.; Brayne, C.; et al. Dementia prevention, intervention, and care: 2024 report of the Lancet standing Commission. *Lancet* **2024**, *404*, 572-628, doi:10.1016/S0140-6736(24)01296-0.
